# Telehealth cognitive behavioural therapy improves health-related quality of life and pain in endometriosis: the Healing Pelvic Pain Intervention (HaPPI)—a randomized controlled trial

**DOI:** 10.1093/hropen/hoag006

**Published:** 2026-01-22

**Authors:** Subhadra Evans, David Skvarc, Adrian Esterman, Matthew I Mackay, Melissa O’Shea, Leesa Van Niekerk, Marilla L Druitt, Jim Tsaltas, Simon R Knowles, Elesha Parigi, Katherine Stanley, Jill Harris, Meg Barber, Madeleine Dober, Charlotte Dowding, Antonina Mikocka-Walus

**Affiliations:** School of Psychology, Deakin University, Geelong, VIC, Australia; School of Psychology, Deakin University, Geelong, VIC, Australia; School of Public Health, College of Health, Adelaide University, Adelaide SA, Australia; School of Psychology, Deakin University, Geelong, VIC, Australia; School of Psychology, Deakin University, Geelong, VIC, Australia; School of Psychological Sciences, University of Tasmania, Hobart, TAS, Australia; Barwon Health (University Hospital), Geelong, VIC, Australia; School of Medicine, Deakin University, Geelong, VIC, Australia; Department of Gynaecology, Epworth Freemasons Hospital, Melbourne, VIC, Australia; Department of Obstetrics and Gynaecology, Monash Health and Monash University, Melbourne, VIC, Australia; Department of Psychological Sciences, Faculty of Health, Arts and Design, Swinburne University of Technology, Melbourne, VIC, Australia; School of Psychology, Deakin University, Geelong, VIC, Australia; School of Psychology, Deakin University, Geelong, VIC, Australia; Kyo Yoga & Healing, Ocean Grove, VIC, Australia; Barwon Health (University Hospital), Geelong, VIC, Australia; The Royal Melbourne Hospital, Melbourne, VIC, Australia; School of Psychology, Deakin University, Geelong, VIC, Australia; Orange Health Service, Orange, NSW, Australia; School of Psychology, Deakin University, Geelong, VIC, Australia

**Keywords:** endometriosis, quality of life, cognitive behavioural therapy, yoga, telehealth, randomized controlled trial

## Abstract

**STUDY QUESTION:**

Is telehealth cognitive behavioural therapy (CBT) or yoga effective in improving health-related quality of life (HRQoL) and secondary outcomes for endometriosis?

**SUMMARY ANSWER:**

Endometriosis-tailored CBT is superior to education for improving HRQoL and pain.

**WHAT IS KNOWN ALREADY:**

Endometriosis is a burdensome disease that contributes to diminished quality of life. Current biomedical care including hormonal and analgesic treatment is associated with inconsistent efficacy. Interdisciplinary care is therefore needed to augment the well-being of people with endometriosis. Although studies have suggested that CBT and yoga are promising for relieving pain and other symptoms associated with endometriosis, the evidence remains limited because they are based on small pilot studies.

**STUDY DESIGN, SIZE, DURATION:**

In this parallel 8-week randomized controlled trial (RCT), 334 participants were randomized using a computer-generated sequence with allocation concealment between April 2021 and February 2024. Outcome assessors were blinded to the group.

**PARTICIPANTS/MATERIALS, SETTING, METHODS:**

Participants required a diagnosis of endometriosis with pain for at least 6 months, and access to internet. Participants were randomly allocated to: (i) CBT (8-week telehealth therapist-led group, n = 79); (ii) yoga (8-week telehealth therapist-led group, n = 83); or (iii) education materials via email (n = 84). Primary outcomes: endometriosis-specific HRQoL (Endometriosis Health Profile-30; EHP-30 Total and Pain); general HRQoL (EQ-5D-5L global health) at post-treatment (8 weeks). Secondary outcomes: pain (period pain, bowel pain, bladder pain, and sexual pain), pain catastrophizing, pain self-efficacy, psychological distress, sleep, fatigue, menstrual symptoms, and central sensitization. Mixed-effects models examined group by time differences.

**MAIN RESULTS AND THE ROLE OF CHANCE:**

The CBT group reported statistically significant improvements in endometriosis-specific HRQoL (EHP Pain *β* = −0.58, 95% CI = −0.89, −0.26, *P* = 0.01), and general HRQoL (EQ-5D-5L global health *β* = 0.52, 95% CI = 0.21, 0.84, *P* = 0.02) compared to the education control group. CBT was also superior to education for pelvic pain (menstrual pain, bowel pain, bladder pain, and sexual pain), pain self-efficacy, and pain catastrophizing. Effect sizes for CBT were generally medium to large (Cohen’s D = −0.28 to −0.93). Yoga was superior to education for menstrual symptoms and sexual pain, with medium effect sizes (Cohen’s D = −0.56 to −0.71).

**LIMITATIONS, REASONS FOR CAUTION:**

Due to the presence of COVID-19 restrictions during data collection, CBT and yoga were delivered online. As a result, it is unclear whether CBT and yoga would have the same effects if delivered face-to-face, or whether online delivery would show comparable efficacy in a post-pandemic context.

**WIDER IMPLICATIONS OF THE FINDINGS:**

Our RCT is the first to test the efficacy of telehealth CBT or yoga for improving outcomes in people living with endometriosis compared to an active control. Telehealth CBT demonstrated efficacy for improving endometriosis-specific and general HRQoL in people with endometriosis, as well as pain outcomes. Yoga demonstrated efficacy for improving menstrual symptoms and pain during sex. We recommend endometriosis-tailored CBT as part of interdisciplinary management for people with endometriosis, including online delivery to address access and mobility barriers. Yoga may be helpful in augmenting pelvic health.

**STUDY FUNDING/COMPETING INTEREST(S):**

This work is supported by the Australian Government, Canberra under the Medical Research Future Fund grant number MRFF1200214. The authors do not have any conflicts of interest in relation to the present study.

**TRIAL REGISTRATION NUMBER:**

ACTRN12620000756921 https://www.anzctr.org.au/Trial/Registration/TrialReview.aspx?id=379947&isReview=true.

**TRIAL REGISTRATION DATE:**

22 July 2020.

**DATE OF FIRST PATIENT’S ENROLMENT:**

23 April 2021.

WHAT DOES THIS MEAN FOR PATIENTS? Our randomized controlled trial (n = 334) comparing online cognitive behavioural therapy (CBT) or yoga to a control group sent education materials via email is the first to test the efficacy of these interventions for improving outcomes in people living with endometriosis. It is also the first study to test an endometriosis-specific CBT protocol delivered online, showing CBT is superior to an active control in improving health-related quality of life and pain. Given that one in nine reproductive-aged women is impacted by endometriosis, evidence based digital interventions are crucial for scalability and implementation. The results are likely to be particularly beneficial for people living in areas with limited access to specialized care.

## Introduction

Endometriosis is a burdensome disease, impacting one in nine reproductive-aged women and costing more than $7.7 billion in Australia alone per year (Australian Government Department of Health, 2018). Although symptoms vary, pelvic pain and impaired health-related quality of life (HRQoL) are common (Jia *et al*., 2012; Gambadauro *et al*., 2019). Biomedical care, involving surgery, hormones, and pain medication, is associated with inconsistent efficacy, side effects, and medication discontinuation in 10 to 40% of patients (Sinaii *et al*., 2007). As outlined by the Royal Australian and New Zealand College of Obstetricians and Gynaecologists (RANZCOG, 2021), there is a need for interdisciplinary, supportive care to augment the well-being of people with endometriosis (Australian Government Department of Health, 2018). In particular, psychological interventions can address the emotional aspects of living with a chronic health condition, while mind–body interventions such as yoga may be beneficial via stress reduction and engagement in movement. The delivery of such interventions with digital technology presents an opportunity to broaden access to previously underserved patients (Perelmuter and Shin, 2025).

Cognitive behavioural therapy (CBT), an established psychological treatment for chronic pain (Ehde *et al*., 2014), involves identifying and changing unhelpful thoughts and behaviours, with techniques for coping and re-engagement in life tasks (Butler *et al*., 2006). A recent randomized controlled trial (RCT) comparing CBT (n = 25) to a usual care control group (n = 27) for women with endometriosis and pain found improved HRQoL, pain, and mental health in the CBT group (Donatti *et al*., 2025). Yoga, involving mindful movement, breath regulation exercises, and meditation, improves pain (Posadzki *et al*., 2011) and mental health (de Manincor *et al*., 2016), with supportive pilot findings for endometriosis (Gonçalves *et al*., 2017). While promising, such studies are under-powered and require appropriate controls to test efficacy (Evans *et al*., 2019).

There is a need to use innovative technology to reduce access barriers; in particular, skilled psychologists and yoga therapists are often unavailable in rural and remote communities. Given that virtually-delivered CBT and yoga are helpful for mental health and pain in other populations (Luo *et al*., 2020; Esfandiari *et al*., 2021; Tankha *et al*., 2024), and represent a low-cost and scalable model compared to individual face-to-face therapy, an important research goal is to test virtually-delivered group CBT and yoga for endometriosis, compared to an active control. The present study examined the efficacy of group telehealth (virtual therapist-delivered) CBT and yoga on endometriosis HRQoL and secondary outcomes (pain, psychological distress, sleep, fatigue, menstrual symptoms, pain catastrophizing, pain self-efficacy, and central sensitization) compared to an active control. It was hypothesized that, compared to education, participants randomized to telehealth CBT or yoga would show significant post-intervention HRQoL and secondary outcome improvements at 8 weeks.

## Materials and methods

### Design

A single-blinded parallel RCT, where participants were randomly allocated to yoga, CBT, or education (1:1:1 ratio). The study was advertised as a trial comparing ‘mind–body interventions’. Randomization occurred after participants signed consent and before completion of the baseline questionnaires using a computerized randomization schedule and allowing for allocation concealment. Random function in Excel was used to generate an allocation list in permutation blocks of 6. Participants were enrolled and assigned to groups by the trial manager. Outcome measures were collected via online self-report questionnaires at baseline, post-intervention (8 weeks) as well as 6- and 12-month follow-ups. The findings for the present study focus on change post-intervention (8 weeks), with a future planned follow-up and health economics analysis. The statisticians performing the analyses were blinded to group status. Participants in all groups continued their usual medical care.

### Ethical approval and trial registration

The protocol was approved by Barwon Health Research Ethics Committee and Monash Health in November 2020 (Ref. 65948 and RES-20-0000-838X). Written informed consent was obtained before participants were randomized to the groups. The trial was prospectively registered in the Australian New Zealand Trial Registry on 22 July 2020 (ID: ACTRN12620000756921p). Further detail about the methods is available elsewhere (Mikocka-Walus *et al*., 2021)^.^

### Participants

#### Inclusion criteria

Inclusion criteria included: (i) diagnosis of endometriosis with pelvic pain for at least 6 months, supported by an ultrasound, histology, surgical report, or letter from treating physician; (ii) at least 18 years of age; (iii) capacity to provide informed consent; (iv) sufficient English to answer questionnaires, and engage with the intervention and group; and (v) access to internet.

#### Exclusion criteria

Exclusion criteria included: (i) high risk of harming self or others, current severe mental illness (e.g. schizophrenia, severe depression), or significant cognitive impairment as confirmed during psychological screening by clinical psychology team; (ii) major physical issues/injuries; (iii) currently pregnant; and (iv) recent therapist-led course of CBT or yoga (within the past 6 months)

### Recruitment

Potential participants were recruited via clinician offices and social media (e.g. advertising on Instagram and websites of: EndoHelp Foundation; Endozone; Endometriosis Australia). Interested individuals contacted the trial manager to express their interest in participating in the study. Individuals were required to provide a letter from their treating physician confirming their diagnosis. After participants completed the online consent form, they were assessed by a registered or provisional psychologist for relevant psychological exclusions.

### Sample size

We estimated an effect size for a repeated-measure ANOVA, with three groups, and four time-points (only baseline and immediately post-intervention are reported in the present study) in R (R Foundation for Statistical Computing, Vienna, Austria), using the WebPower package (Zhang and Mai, 2023). The power analysis was conducted using an earlier version of R, but the results are reproducible under the current version (4.4.2). With an alpha of 0.01, 80% power, and an effect size of *f* = 0.25, the minimum sample size for the entire sample is N = 226 or n = 76 per group. Accounting for 15% dropout rates over the duration of the intervention, the minimum total sample size for this study was set at N = 259 (86 per group). However, the 15% dropout rate was an estimate based on the effects of in-person interventions (Swift and Greenberg, 2012); there were a limited number of empirical studies investigating the efficacy of online-delivered CBT and yoga for endometriosis.

### Interventions

Initially, the trial was designed to include face-to-face or telehealth options, depending on Covid restrictions (Mikocka-Walus *et al*., 2021). The trial began while restrictions were in place in Victoria, Australia (lockdowns and strict restrictions ran from March 2020 to October 2022). Given positive feedback from participants regarding the convenience of telehealth CBT and yoga, and reluctance of participants to engage in groups due to fear of infection directly following restrictions, the trial remained telehealth. Eleven CBT cohorts, 10 yoga cohorts, and 9 education cohorts were run. Groups were capped at 13 participants to ensure sufficient support from therapists. All groups continued with their usual medical care during the trial and received well-being check-in phone calls from study research staff at the end of weeks 1, 4, and 8.

#### CBT

Weekly group CBT sessions of 120 minutes for 8 weeks, delivered via Zoom. Study psychologists and a consumer advocate (M.D., K.S., M.B., C.D.) adapted Thorn’s Cognitive Therapy for Chronic Pain protocol to include examples relevant for people with endometriosis and pelvic pain (Thorn, 2017). The programme aims to assist individuals to gain: an understanding of pain including chronic pain education; practical cognitive and behavioural strategies for managing pain and mood including mindfulness; and experience practicing strategies both in and outside the group. Participants were encouraged to complete homework tasks such as thought diaries and guided relaxation sessions (approximately 20 minutes of home practice) at least 3 days per week. Three psychologists (M.D., M.B., C.D.) experienced in working with people with chronic pelvic pain delivered the CBT intervention.

#### Yoga

Weekly group yoga sessions of 60 minutes for 8 weeks, delivered via Zoom. Classes included physical postures (suitable for all levels of experience); breath awareness and techniques; and relaxation and meditation. The yoga sequence was developed into a protocol, based on the therapeutic yoga approach of T. Krishnamacharya, which emphasizes adaptations for individual needs and capacities (Desikachar, 1999). Before beginning group classes, participants attended a one-on-one 60-minute online session with the yoga therapist to develop a personalized home practice, accounting for any injuries or health issues. Participants were encouraged to complete the home practice (approximately 15 to 20 minutes) at least 3 days per week. Each home practice incorporated simple movement and breathing techniques and was designed to complement group classes. The intervention was delivered and overseen by a senior yoga therapist (a yoga therapist receives additional training to tailor practices for specific physical and/or mental health needs), who is a senior mentor with Yoga Australia with experience in delivering yoga practices for people with chronic pain.

#### Education (control)

The control group received education via weekly emails from the trial manager, consisting of eight endometriosis education handouts, related to topics such as symptoms and causes; diagnosis; management; fertility and pregnancy; relationships and emotions. The education materials provided to participants were adapted from materials developed by Jean Hails for Women’s Health, a not-for-profit organization dedicated to improving women’s health. Education was chosen as the active control because it is the standard support often provided to patients. Additionally, health education control groups are recommended when testing the efficacy of psychological therapy, to account for the non-specific benefits of engaging in therapy (Ymer *et al*., 2022).

#### Intervention fidelity

Treatment fidelity to yoga was maintained by adopting the same yoga sequence across classes, delivered by a trained and qualified yoga therapist. Nine of the 10 yoga cohorts were delivered by the same senior yoga therapist, who provided oversight of the additional cohort they did not directly teach. Treatment fidelity to CBT was maintained by using a consistent therapy protocol across psychologists experienced in CBT. In addition, 25 (28%) CBT sessions were recorded and monitored for fidelity checks; 91.33% of the therapy protocol was adhered to across the 25 recorded sessions.

### Primary outcomes

#### Endometriosis-related QoL

The Endometriosis Health Profile (EHP-30), a patient-reported outcome, which exhibits good reliability and validity (Bourdel *et al*., 2019), was used to measure patient’s perspective about the impact of endometriosis. The scale includes domains related to pain, control, emotional well-being, social support, and self-image (summed to produce an overall EHP Total score). The EHP Total score, as well as EHP Pain scores are recommended for use in endometriosis clinical trials (Vincent *et al*., 2010), and thus, the EHP Total and EHP Pain subscale were included in the present study. The EHP Pain subscale measures the impact of pain on various aspects of life, including daily activities, mood, and overall well-being. Recall period is the last 4 weeks, with each domain ranging from 0 (best possible health) to 100 (worst). Reliability estimates (i.e. measure of internal consistency) for EHP Total and EHP Pain subscale at baseline (*α* = 0.95) and week 8 were excellent (*α_range_* = 0.93 to 0.96).

#### General QoL

Measured by the EQ-5D-5L, the overall health visual analogue scale (VAS) was used, from 0 (worst possible health) to 100 (best) as a measure of global HRQoL. The utility score encompassing mobility, self-care, usual activities, pain/discomfort, and mental health was reserved for an associated health economics analysis. The EQ-5D-5L has good reliability and validity (Janssen *et al*., 2013).

### Secondary outcomes

#### Pelvic pain

Measured with a series of 11-point numeric rating scales (0 = no pain, 10 = worst pain possible) asking about usual level of pain (without pain medication) related to: menstruation, use of bowels, use of bladder, and sexual activity. Pain numeric rating scales show reliability and validity in measuring pain severity (Alfonsin *et al*., 2019).

#### Pain self-efficacy scale

Participants answered 10 items about their confidence in coping with pain from endometriosis in their daily lives (e.g. ‘I can cope with my pain in most situations’) on a 7-point Likert scale (0 = not at all confident, 6 = completely confident). Scores were summed together to create an overall pain self-efficacy score and showed excellent reliability at baseline (*α* = 0.93) and week 8 (*α* = 0.94).

#### Pain catastrophizing scale

Participants answered 13 items assessing rumination, magnification, and feeling helpless about pain (e.g. ‘I feel I can’t go on’) on a 5-point Likert scale (0 = not at all, 4 = all the time). Scores were summed together to create an overall pain catastrophizing score and showed excellent reliability at baseline (*α* = 0.90) and week 8 (*α* = 0.91).

#### Menstrual symptoms questionnaire

Includes nine symptoms using a scale from 0 (not present) to 10 (extremely severe); abdominal cramps, dull abdominal pain or discomfort, low back pain, headache or migraine, aches all over, bloating, nausea, diarrhea, and bowel movements (Chen *et al*., 2018). Scores were summed, with higher scores indicating worse menstrual symptoms. Reliability estimates at baseline were good (*α* = 0.74) and excellent at week 8 (*α* = 0.80).

#### Central sensitization

Measured using the Fibromyalgia Criteria-2016—a proxy indicator for central sensitization (Wolfe *et al*., 2016). Participants responded to six items assessing somatic symptoms and a question about pain sites, with scores summed to create a polysymptomatic distress scale (0–31). Baseline (*α* = 0.81) and week 8 (*α* = 0.86) were excellent.

#### Jenkins Sleep Scale (JSS)

A self-reported scale for individuals with chronic pain. Participants responded to four items (e.g. ‘Have trouble falling asleep’) on a 6-point Likert scale (0 = not at all; 5 = 22–30 days) (Jenkins et al., 1988). Scores were summed, with higher scores indicating worse sleep quality and showed good reliability to baseline (*α* = 0.71) and week 8 (*α* = 0.78).

#### Fatigue Symptom Inventory—disruption subscale (FSI)

Participants responded to four items (e.g. ‘fatigue interfered with your ability to bathe and dress yourself’) on an 11-point Likert scale (0 = no interference; 10 = extreme interference) (Donovan and Jacobson, 2010). Scores were summed, with higher scores indicating extreme interference from fatigue, with reliability estimates at baseline (*α* = 0.91) and week 8 (*α* = 0.93) indicating excellent reliability.

#### Psychological distress scale (DASS-21)

Measured by summing items relating to anxiety, depression, and stress symptoms (e.g. ‘I found it hard to wind down’) which participants responded to on a 4-point Likert scale (0 = did not apply to me at all; 4 = applied to me very much, or most of the time) (Henry and Crawford, 2005). Scores were summed, with higher scores indicating higher psychological distress. Reliability estimates were excellent at baseline (*α* = 0.92) and week 8 (*α* = 0.94).

#### Adverse events

All participants were contacted at the end of weeks 1, 4, and 8, for well-being checks and to discuss any event(s) that may have occurred in the context of their participation in the allocated intervention. Participants were also informed they could email outside the scheduled well-being checks if necessary. Any events were evaluated by the trial manager with the participant for severity (*mild, moderate, severe, whether expected*) and whether related to the intervention (*related, not related*).

#### Satisfaction

Measured at week 8 with the item ‘How satisfied are you with the mind–body intervention during the past 8 weeks?’ from 1 (*not at all satisfied*) to 4 (*very satisfied*).

### Statistical analysis

**Figure 1.**
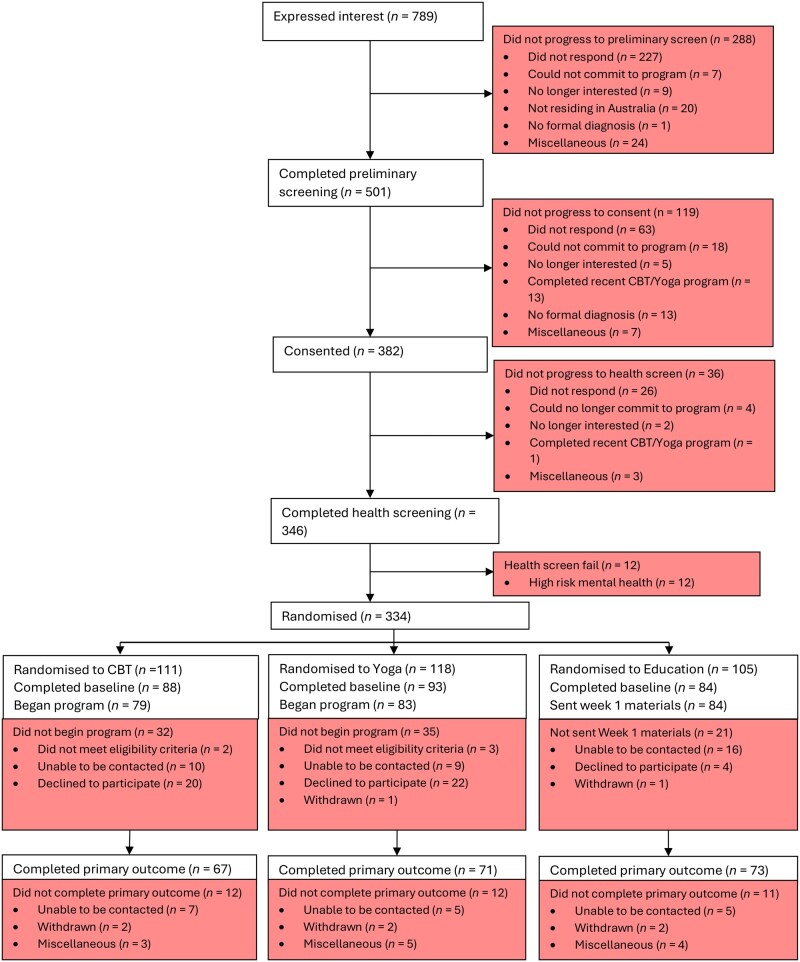
**HaPPI study CONSORT diagram to primary outcome data**.

A CONSORT statement diagram is provided (Fig. 1) showing the flow of subjects through the trial (Schulz *et al*., 2010). Using Stata 18 (StataCorp LLC, College Station, TX, USA), we used linear mixed effects models to assess changes between baseline and post-intervention for each outcome measure. Each model comprised the outcome measure as the dependent variable, fixed effects of time, intervention group, and a group-time interaction term, with the subject ID as the random effect. Baseline values for each outcome variables were included in each model, and the overall fit of each model was assessed with Wald’s chi-square tests. To compare the relatively efficacy of CBT and yoga against education, we calculated standardized mean differences effect sizes and 95% confidence intervals calculated from the pooled variance of each model. We observed no discernible pattern of missing data. An ANOVA was conducted to determine whether mean satisfaction with the intervention scores were significantly different between groups. As a post-hoc analysis, we examined the proportion of participants who met the minimal clinically important difference (MCID) values for the primary outcomes (EHP Pain, EHP Total, and EQ-5D-5L). There are no established MCID levels, with estimates for EHP Pain between 11.5 and 30 (Jones *et al*., 2004; van de Burgt *et al*., 2013; Pokrzywinski *et al*., 2020). For EHP Total, we followed the same estimates. A systematic review on MCID for EQ-5D-5L indicates it is between 7.5 and 8.4 (Cheng *et al*., 2024).

## Results

Participant flow is shown in Fig. 1. A total of 789 people expressed interest in the study, with 334 randomized; 79 began CBT, 83 began yoga, and 84 were sent education materials. Primary outcome data were available for 211 participants. Follow-up dropout rates from the point of randomization to post-treatment were 40%, 40%, and 30% for CBT, yoga, and education, respectively (from randomization to beginning treatment: 29%, 30%, and 20% for CBT, yoga, and education, respectively). Participants allocated to CBT and yoga had twice the odds of dropout compared to those in education; OR = 2.08 [1.18–3.36] and OR = 2.05 [1.17–3.61]. Although our estimate of oversampling by 15% to account for participant dropout was lower than our actual attrition rates, we still had sufficient power to detect significant effects and determine intervention efficacy. As shown in Table 1, the average participant in all groups was in their mid-30s, Australian-born, university educated, without children, and had a moderate level of pain.

**Table 1.** Participant sociodemographic characteristics.

	Cognitive behavioural therapy	Yoga	Education
**Age**	33.4 ± 8.1	34.1 ± 7.5	30.2 ± 7.8
**Sex assigned at birth**			
Female	87 (98.9)	92 (98.9)	84 (100)
Male	1 (1.1)	1 (1.1)	0 (0)
**Gender identity**			
Woman	86 (97.7)	93 (100.0)	83 (98.8)
Man	0 (0)	0 (0)	1 (1.2)
Non-binary	2 (2.3)	0 (0)	0 (0)
**Aboriginal or Torres Strait Islander**	0 (0)	0 (0)	2 (2.4)
**Level of education obtained**			
Less than year 12	0 (0)	1 (1.1)	2 (2.4)
Year 12 or equivalent	13 (14.8)	11 (11.8)	16 (19.0)
Vocational education (TAFE)	20 (22.7)	17 (18.3)	18 (21.4)
Bachelor’s degree	34 (38.6)	39 (41.9)	29 (34.5)
Postgraduate degree	13 (14.8)	20 (21.5)	13 (15.5)
**Location**			
Metro	70 (79.5)	66 (71.0)	68 (81.0)
Inner regional	15 (17.0)	25 (26.9)	13 (15.5)
Outer regional	2 (2.3)	2 (2.2)	3 (3.57)
**Relationship status**			
Single (never married)	31 (35.2)	36 (38.7)	28 (33.3)
Married/de facto	54 (61.4)	55 (59.1)	49 (58.3)
Widowed	0 (0)	0 (0)	1 (1.2)
Divorced	2 (2.3)	2 (2.2)	3 (3.6)
**Work status**			
Employed full time	49 (55.7)	42 (45.2)	37 (44.0)
Employed part time	18 (20.5)	27 (29.0)	24 (28.6)
Unemployed	3 (3.4)	4 (4.3)	4 (4.8)
Student	6 (6.8)	4 (4.3)	8 (9.5)
Homemaker	2 (2.3)	1 (1.1)	1 (1.2)
Self-employed	2 (2.3)	8 (8.6)	4 (4.8)
Unable to work	2 (2.3)	3 (3.2)	2 (2.4)
**Country of birth**			
Australia	67 (76.1)	77 (82.8)	66 (78.6)
Other	21 (23.9)	16 (17.3)	18 (21.4)
**Language spoken**			
English	85 (96.6)	90 (96.8)	84 (100)
Other	3 (3.4)	3 (3.23)	0 (0)
**Children**			
Yes	25 (28.4)	27 (29.0)	15 (17.9)
**Smoking status**			
Current smoker	6 (6.8)	1 (5.4)	5 (6.0)
Former smoker	26 (29.5)	18 (19.4)	18 (21.4)
Never smoked	56 (63.6)	70 (75.3)	61 (72.6)
**Alcohol use**			
More than two standard drinks per day	0 (0)	2 (2.2)	1 (1.2)
Fewer than two drinks per day	4 (4.5)	5 (5.4)	1 (1.2)
Drinking several times a week	7 (8.0)	7 (7.5)	10 (11.9)
Occasional drinking	61 (69.3)	64 (68.8)	57 (67.9)
Never drinking	16 (18.2)	15 (16.1)	15 (17.9)
**Usual level of pain during period**	7.3 ± 1.71	7.4 ± 1.83	7.2 ± 1.74

Values are mean ± SD or *n* (%).

Examination of participant dropout revealed no association between dropout and demographic factors, education level, marital status, employment, or recruitment cohort. However, we observed that completing participants reported significantly lower EHP Pain at baseline compared to dropouts (MD = 7.69, Welch’s (1, 101) = 10.1, *P* = 0.002), and greater pain self-efficacy (MD = 4.04, F (1, 261) = 4.32, *P* = 0.039). As shown in Table 2, attendance for CBT was higher in weeks 1 and 4, and home practice higher in week 2, compared to yoga. Attendance for both CBT and yoga declined over time.

**Table 2.** Weekly cognitive behavioural therapy and yoga attendance and home practice.

Attendance of sessions						Homework				
**Week**	**Cognitive behavioural therapy**		**Yoga**		**Comparison**	**Cognitive behavioural therapy**		**Yoga**		**Comparison**
	*n*	**%**	*n*	**%**		*n*	**%**	*n*	**%**	
1	64	95.5	54	77.1	**χ^2^_1_ = 9.68, *P* = 0.00**	52	77.6	48	68.6	χ^2^_1_ = 1.41, *P* = 0.32
2	55	82.1	48	68.6	χ^2^_1_ = 3.35, *P* = 0.10	53	79.1	43	61.4	**χ^2^_1_ = 5.10, *P* = 0.04**
3	53	79.1	50	71.4	χ^2^_1_ = 1.08, *P* = 0.40	42	62.7	44	62.9	χ^2^_1_ = 0.00, *P *= 1.00
4	55	82.1	43	61.4	**χ^2^_1_ = 7.18, *P* = 0.01**	38	56.7	35	50	χ^2^_1_ = 0.62, *P* = 0.54
5	50	74.6	44	62.9	χ^2^_1_ = 2.20, *P* = 0.19	34	50.7	39	55.7	χ^2^_1_ = 0.34, *P* = 0.68
6	42	62.7	35	50	χ^2^_1_ = 2.24, *P* = 0.19	31	46.3	33	47.1	χ^2^_1_ = 0.01, *P *= 1.00
7	43	64.2	33	47.1	χ^2^_1_ = 4.02, *P* = 0.07	33	49.3	32	45.7	χ^2^_1_ = 0.17, *P* = 0.81
8	46	68.7	38	54.3	χ^2^_1_ = 2.98, *P* = 0.12	24	35.8	20	28.6	χ^2^_1_ = 0.83, *P* = 0.47

Chi-square tests of independence were conducted to compare session attendance and homework completion between cognitive behavioural therapy and yoga. *n*: number of participants. Comparisons in bold indicate a significant group difference.

### Primary outcomes

Descriptive statistics for continuous and categorical outcomes are presented in Table 3. Results of linear mixed effects models are shown in Table 4. The CBT group reported statistically significant improvements in endometriosis-specific HRQoL (EHP Pain B = −5.83, SE = 2.29, *P* = 0.01), which equates to a 21% improvement; and general HRQoL (EQ-5D-5L global health B = 6.95, SE = 3.01, *P* = 0.02) compared to the control group, with medium effect sizes.

**Table 3.** Descriptive statistics for intervention groups over time.

		Baseline			Post-intervention		
	**Intervention**	***N***	***M***	***SD***	***N***	***M***	***SD***
**Primary outcomes**							
EHP 30 total	Cognitive behavioural therapy	88	53.6	20.1	67	44.8	19.9
	Education	84	55.1	14.7	73	51.3	17.3
	Yoga	93	50.1	16.4	71	45.0	21.1
EHP 30 pain	Cognitive behavioural therapy	88	49.56	20.43	67	39.32	19.72
	Education	84	48.21	16.91	73	44.55	17.46
	Yoga	93	45.97	17.59	71	41.26	20.41
QoL Global Health	Cognitive behavioural therapy	88	59.0	19.5	67	64.3	19.8
	Education	84	62.5	19.1	73	60.3	21.3
	Yoga	93	60.1	18.1	70	60.9	21.0
**Secondary outcomes**							
Period pain	Cognitive behavioural therapy	68	7.3	1.7	63	6.6	2.3
	Education	59	7.2	1.8	71	7.1	1.9
	Yoga	74	7.4	1.8	69	6.6	2.4
Bowel pain	Cognitive behavioural therapy	88	4.4	2.7	67	3.34	2.6
	Education	84	3.6	2.8	73	3.5	2.8
	Yoga	93	3.9	2.6	71	3.5	2.6
Bladder pain	Cognitive behavioural therapy	88	2.3	2.4	67	1.8	2.2
	Education	84	2.1	2.4	74	2.3	2.8
	Yoga	93	1.7	2.2	71	1.7	2.2
Painful sex	Cognitive behavioural therapy	86	4.9	2.8	65	4.4	2.8
	Education	84	4.6	2.7	74	4.4	2.6
	Yoga	91	4.6	2.6	69	3.8	2.4
Menstrual symptoms	Cognitive behavioural therapy	68	51.9	13.2	53	47.7	15.7
	Education	58	51.6	13.1	50	48.6	13.9
	Yoga	73	52.9	14.2	53	45.0	15.6
Pain catastrophizing	Cognitive behavioural therapy	88	23.1	12.30	66	18.3	12.8
	Education	83	23.9	12.64	72	23.5	12.6
	Yoga	92	19.8	11.43	67	19.0	11.2
Central sensitization	Cognitive behavioural therapy	88	14.9	5.3	67	13.6	6.3
	Education	84	14.4	4.7	75	13.8	5.5
	Yoga	93	14.2	4.7	71	12.5	5.8
Psychological distress	Cognitive behavioural therapy	88	39.9	27.51	66	35.1	25.6
	Education	83	37.9	19.51	72	35.6	24.0
	Yoga	92	35.2	19.57	68	31.4	20.9
Pain self-efficacy	Cognitive behavioural therapy	88	33.5	13.21	66	38.5	13.6
	Education	83	33.0	11.87	72	33.2	12.00
	Yoga	92	35.5	12.96	68	37.3	13.6
Sleep	Cognitive behavioural therapy	88	11.3	4.4	67	10.5	5.2
	Education	84	10.4	4.6	72	10.1	4.8
	Yoga	93	10.8	4.4	70	9.2	4.82
Fatigue (disruption)	Cognitive behavioural therapy	88	34.1	14.2	66	31.6	16.1
	Education	83	34.2	14.3	73	29.9	13.5
	Yoga	93	31.1	16.0	70	27.1	15.6

*N*: number of participants; *M*: mean; *SD*: standard deviation; EHP: endometriosis health profile; QoL: quality of life.

**Table 4.** Intervention groups versus education over time, with effect sizes.

	Yoga vs education over time		Cognitive behavioural therapy vs education over time	
	Unstandardized	StandardizedES [95% CI]	Unstandardized	StandardizedES [95% CI]
**Primary outcomes**				
EHP Pain	*B* = −1.11, SE = 2.26, *z* = −0.49*, P *= 0.62	−0.11 [0.42, 0.2]	***B* = **−**5.83, SE = 2.29, *z* = **−**2.54, *P *= 0.01**	−**0.58 [**−**0.89,** −**0.26]**
EHP Total	*B* = −0.82, SE = 2.2, *z* = −0.41, *P *= 0.68	−0.09 [−0.4, 0.22]	*B* = −3.64, SE = 2.05, *z* = −1.77, *P *= 0.08	−0.4 [−0.72, −0.08]
QoL Global Health	*B* = 3.28, SE = 2.96, *z* = 1.11, *P *= 0.26	0.25 [−0.06, 0.56]	***B* = 6.95, SE = 3.01, *z* = 2.31, *P *= 0.02**	**0.52 [0.21, 0.84]**
**Secondary outcomes**				
Period pain	*B* = −0.48, SE = 0.24, *z* = −1.94, *P *= 0.05	−**0.5 [**−**0.85,** −**0.15]**	***B* = **−**0.89, SE = 0.25, *z* = **−**3.54, *P* = < 0.001**	−**0.93 [**−**1.29,** −**0.57]**
Bowel pain	*B* = −0.32, SE = 0.32, *z* = −1.00, *P *= 0.32	−0.22 [−0.53, 0.09]	***B* = **−**0.69, SE = 0.32 *z* = **−**2.1, *P *= 0.04**	−**0.47 [**−**0.79,** −**0.15]**
Bladder pain	*B* = −0.32, SE = 0.26, *z* = −1.2, *P *= 0.23	−0.27 [−0.58, 0.04]	***B* = **−**0.71, SE = 0.26, *z* = **−**2.65, *P *= 0.002**	−**0.6 [**−**0.92,** −**0.28]**
Painful sex	***B* = **−**0.74, SE = 0.29, *z* = **−**2.51, *P *= 0.01**	−**0.56 [**−**0.88,** −**0.25]**	***B* = **−**0.39, SE = 0.29, *z* = **−**2.51, *P *= 0.01**	−0.3 [−0.62, 0.02]
Menstrual symptoms	***B* = **−**5.27, SE = 1.97, *z* = **−**2.67, *P *= 0.01**	−**0.71, [**−**1.07,** −**0.35]**	*B* = −2.59, SE = 1.99, *z* = −1.3, *P *= 0.19	−0.35 [−0.72, 0.02]
Pain catastrophizing	*B* = 0.02, SE = 1.34, *z* = 0.01, *P *= 0.99	−0.05 [−0.21, 0.12]	***B* = **−**3.56, SE = 1.36, *z* = **−**2.63, *P *= 0.01**	−**0.28 [**−**0.44,** −**0.11]**
Central sensitization	*B* = 0.06, SE = 0.65, *z* = 0.09, *P *= 0.93	0.02 [−0.28, 0.32]	*B* = −0.18, SE = 0.66, *z* = −0.28, *P *= 0.78	−0.06 [−0.37, 0.25]
Psychological distress	*B* = −0.84, SE = 2.41, *z* = −0.35, *P* = 0.72	−0.24 [−0.56, 0.07]	*B* = 3.41, SE = 2.42, *z* = 1.4, *P* = 0.16	0.01 [−0.31, 0.33]
Pain self-efficacy	*B* = 1.11, SE = 1.6, *z* = 0.70, *P* = 0.49	0.16 [−0.16, 0.47]	***B* = 4.01, SE = 1.61, *z* = 2.48, *P *= 0.01**	**0.56 [0.24, 0.88]**
Sleep	*B* = −1.11, SE = 0.56, *z* = −1.95, *P *= 0.05	−**0.44 [**−**0.75,** −**0.13]**	*B* = −0.20, SE = 0.57, *z* = −0.35, *P *= 0.73	−0.08 [−0.4, 0.24]
Fatigue (disruption)	*B* = 1.28, SE = 1.2, *z* = 0.64, *P *= 0.52	0.14 [−0.17, 0.45]	*B* = 1.79, SE = 2.02, *z* = 0.88, *P *= 0.38	0.2 [−0.12, 0.52]

Simple slopes comparisons were evaluated using Wald tests of the time × group interaction coefficients. 95% confidence intervals are calculated using pooled rather than group-level standard error, and are less conservative. Effects in bold represent significantly superior performance over time compared to education. ES: effect size; SE: standard error; CI: confidence interval; EHP: endometriosis health profile; QoL: quality of life.

### Secondary outcomes

The CBT group reported significantly better improvements in pain (period pain *B* = −0.89, SE = 0.25, *P* < 0.001; bowel pain *B* = −0.69, SE = 0.32, *P* = 0.04; bladder pain *B* = −0.71, SE = 0.26, *P* = 0.002; sexual pain *B* = −0.39, SE = 0.29, *P* = 0.01), pain self-efficacy (*B* = 4.01, SE = 1.61, *P* = 0.01), and pain catastrophizing (*B* = −3.56, SE = 1.36, *P* = 0.01) compared to education, with medium to large effect sizes. The yoga group reported significant improvements in menstrual symptoms (*B* = −5.27, SE = 1.97, *P* = 0.01) and sexual pain (*B* = −0.39, SE = 0.29, *P* = 0.01) compared to education, with medium effect sizes, with trends for improved period pain (*B* = −0.48, SE = 0.24, *P* = 0.05) and sleep (*B* = −1.11, SE = 0.56, *z* = −1.95, *P* = 0.05).

### Adverse events

Participants reported 12 adverse events; all were mild and expected; 6 in the yoga group (5 unrelated to the intervention and 1 unlikely related); 5 in the CBT group (4 possibly related and 1 unrelated to the intervention); and 1 in the education group (not related). The majority of events involved a flare in existing physical or mental health issues; for example, feeling anxious after a group discussion about infertility. Participants were offered additional support from the psychology team.

*Satisfaction* for education (*M *= 2.33, *SD *= 9.49) was significantly lower than for CBT (*M *= 3.36, *SD* = 0.73) and yoga (*M *= 3.48, *SD* = 0.66; *F*(2,201) = 43.99, *P* = < 0.001). There was no statistically significant difference in satisfaction between CBT and yoga.

### MCID (post-hoc analysis)

For *EHP Total*, 7% of the CBT group met a MCID of 30, while 4% and 0% did so in the yoga and education groups, respectively; 40% of the CBT group met a MCID of 11.5, while 34% of the yoga group, and 22% of the education group did so. For *EHP Pain*, 10% of participants in the CBT group met the MCID threshold of 30, while 6% and 3% of participants did so in the yoga and education groups, respectively; 42% of participants in the CBT group met the MCID threshold of 11.5, while 24%, and 21% of participants did so in the yoga and education groups, respectively. For general *HRQoL*, 39% of the CBT group, 41% of the yoga group and 27% of the education group met an MCID threshold of 7.5. Similar proportions were found for meeting an MCID threshold of 8.4.

## Discussion

The present study tested the efficacy of (i) CBT and (ii) yoga, delivered via group telehealth, compared to an education control group, on the primary outcome of HRQoL and secondary outcomes of pain (period pain, bladder pain, bowel pain, sexual pain, pain catastrophizing, and pain self-efficacy), psychological distress, menstrual symptoms, sleep, central sensitization, and fatigue. We found support for CBT on HRQoL and all pain outcomes. Yoga was not efficacious for HRQoL but did show evidence for improving menstrual symptoms and sexual pain. Although interdisciplinary care is recommended to support HRQoL in endometriosis (Australian Government Department of Health, 2018; RANZCOG, 2021), this is the first efficacy trial to examine psychological and mind–body treatments compared to an active control.

Our findings regarding the efficacy of CBT for endometriosis are consistent with recent pilot work in women with endometriosis and chronic pain (Donatti *et al*., 2025), showing improved HRQoL and pain after CBT compared to no-intervention. We extend these results, demonstrating that CBT is efficacious for HRQoL and a range of pain outcomes compared to an active control, and moreover, that CBT works in people with endometriosis when delivered via telehealth. Of interest, CBT not only modified pain severity in a range of organs affected by endometriosis but also improved pain cognitions and confidence. Notably, our CBT protocol was tailored for endometriosis, with examples and material derived from our lived experience research and the expertise of psychologists experienced in treating pelvic pain. As such, our findings support the use of CBT that is adapted for endometriosis. However, exploring the mechanism by which CBT improved HRQoL and pain in future studies would be a valuable contribution to the literature. As the CBT group showed a decrease in pain catastrophizing and an increase in pain self-efficacy, these factors may warrant further investigation as potential mediators between CBT and the improvements in HRQoL and pain.

CBT did not affect mental health symptoms or sleep, although this is consistent with prior health psychology research showing that CBT protocols need to be tailored to specific chronic illness symptoms. For example, general CBT protocols are not helpful in addressing functioning in people with gut conditions; rather, examples and exercises need to include gut-related material to be effective (Windgassen *et al*., 2019). The protocol used in the present study was designed to improve pain and functioning and did not directly address mental health or sleep. Additionally, our inclusion criteria required that participants experience pain but did not require participants to have elevated distress levels. Baseline assessments showed only moderate distress, which may explain why the intervention had no impact on this outcome. However, given the high incidence of anxiety and depression in this population (Friedl *et al*., 2015), future research should examine whether CBT protocols tailored to both pain and mental health are warranted to improve a wider range of symptoms.

Research has demonstrated the promise of yoga for endometriosis, including on HRQoL and pain, although prior studies were underpowered and lacking control groups (Evans *et al*., 2019). Our findings suggest that telehealth yoga may have limited benefit for HRQoL, compared to an active control. It is possible that participants missed the engagement of face-to-face yoga; people with endometriosis experience difficulty with movement-based interventions (Sachs *et al*., 2023), and the physical presence of a yoga instructor may aid motivation and engagement. Similarly, the lack of shared in-person experience with other women with endometriosis may have reduced the psychosocial benefits that contribute to yoga’s effectiveness (Gonçalves *et al*., 2017). Also, for two out of eight sessions, participants had lower attendance in yoga compared to CBT. Although our yoga programme was designed for endometriosis, it may have been perceived as too physically demanding compared to ‘talk’ therapy, limiting its potential effectiveness for the study’s sample, with participants less likely to attend and receive benefits from yoga. Additionally, given the complexity of endometriosis, including differences in pain phenotypes and individual profiles of physical and mental health symptoms, it is possible that the yoga practice was not fully consistent with the needs of our specific sample, contributing to lower attendance. Future research should seek consistent feedback from participants to adjust the yoga programme as needed, thereby preserving its potential effectiveness. Similarly, evaluating how effective a yoga intervention is for participants with particular pain areas, and physical and mental health symptoms can deepen understanding under which conditions it is most effective.

In contrast to our primary outcome (HRQoL), yoga was associated with improvements in secondary outcomes, including menstrual symptoms and sexual pain, supporting the benefit of a yoga protocol specifically designed to relieve pelvic tension and discomfort via conscious awareness of the breath and the inclusion of postures (asana) that promote flexibility in the lower back and pelvis. There were also signals for improved period pain and sleep. Given that the present study was only powered for the primary HRQoL outcomes, future studies should appropriately power for pain and sleep quality to understand the full utility of yoga in managing endometriosis symptoms.

Satisfaction rates were higher for CBT and yoga compared to education, indicating positive responses to the interventions. The 40% CBT dropout rate was consistent with recent CBT trial attrition rates; a meta-analysis of 115 CBT studies reported 16% pre-treatment drop out, rising during treatment to a total 35% drop out rate, which was moderated by delivery environment with e-therapy showing significantly higher dropout rates at 60% (Fernandez *et al*., 2015). In fact, retention in remote psychotherapy trials is as low as 50%, even with incentives (Griffith Fillipo *et al*., 2022). The first half of the study was conducted during the COVID-19 pandemic, which may have affected retention rates. Anecdotally, participants were engaged during lockdowns, and more absent when restrictions lifted. The high proportion of participants living across regional Australia suggests that delivering the interventions via telehealth was successful in attracting participants who would ordinarily not have access to specialized psychologists and yoga therapists. Our finding that 80% of adverse events were unrelated to the interventions suggests CBT and yoga can be delivered safely online. However, future research should compare online CBT and yoga with face-to-face CBT and yoga for endometriosis to determine if the mode of delivery influences outcomes.

It is important to note the decrease in participants completing homework tasks over the course of the 8-week intervention. This decline in treatment adherence is common in digital health interventions, even in those with guidance (i.e. frequent contact between therapists and participants) (Baumeister *et al*., 2014). Although automated prompts and reminders can be used to further improve participant engagement (Baumeister *et al*., 2014), future research exploring the utility of online-delivered CBT and yoga for endometriosis should consider adopting evidence-based behaviour change strategies to improve adherence and retention. Behaviour change is most effective when individual (self-efficacy, knowledge, motivation), social (support), and physical (access to resources) factors are addressed (Stokols, 1996). Evidence-based digital behaviour change strategies that could be explored in future studies of digital CBT and yoga include goal setting, self-monitoring, and increased use of prompts or cues. In particular, regular SMS prompts and clear signals of goal tracking may have increased adherence. Enabling participants to monitor and track their progress, and providing them with feedback on performance, may help increase completion rates (Mair *et al*., 2023).Targeting determinants of adherence, such as emphasizing treatment credibility and efficacy (Gasslander *et al*., 2021), or incentivizing 80% completion of homework tasks with a lottery for those who reach this goal, may also prove effective. These strategies (i.e. self-monitoring, feedback, emphasizing treatment credibility and efficacy, and added incentives) may be especially important for supporting individuals who experience pelvic pain intensity and interference and lower pain self-efficacy, as these characteristics were observed in participants who withdrew versus those who completed the interventions.

Finally, the MCID of the study’s findings should be acknowledged. The CBT group experienced a mean reduction of approximately 10 points on the EHP Pain scale and a mean increase of approximately five points on the general HRQoL. While these changes were statistically significant compared to the control, it falls below the established MCID range (EHP Pain = 11.5–30; HRQoL = 7.5–8.4) (Jones *et al*., 2004; van de Burgt *et al*., 2013; Pokrzywinski *et al*., 2020; Cheng *et al*., 2024). However, when using the less stringent MCID for EHP Pain, 42% of the CBT group experienced a MCID. This suggests that heterogeneous subgroups may exist for whom the effectiveness of the intervention may have been substantially different. We intend to investigate the possibility further through response analysis (Hiller *et al.*, 2012), and if such groups are identified, explore meaningful characteristics of those groups.

## Conclusion

This clinical trial meets the need for innovative, high-quality research to improve endometriosis HRQoL (Friedl *et al*., 2015). Although CBT is standard treatment for chronic pain, its efficacy for endometriosis and pelvic pain was yet to be established. This is the first RCT testing CBT for endometriosis, and yoga for endometriosis utilizing an active control, and delivered online. Our findings indicate that even when compared to education (involving non-specific benefits of attention and expectation), HRQoL and pain severity and cognitions are improved with telehealth CBT. These findings have implications for scalability and implementation, as a single therapist in a metropolitan area can provide telehealth CBT to groups nationwide, thereby improving access for people in remote communities and those too unwell to leave home. Furthermore, the provision of group-based therapy may be financially viable, which is pertinent given the high healthcare needs and costs associated with endometriosis (Armour *et al*., 2022). This trial represents a timely step in supporting the interdisciplinary care and HRQoL of people with endometriosis, including those unable to access in-person care.

## Data Availability

The data underlying this article cannot be shared publicly to protect the privacy of the individuals who participated in the study. The data will be shared on reasonable request to the corresponding author.
