# Correction to: Telehealth cognitive behavioural therapy improves health-related quality of life and pain in endometriosis: the Healing Pelvic Pain Intervention (HaPPI)—a randomized controlled trial

**DOI:** 10.1093/hropen/hoag059

**Published:** 2026-06-27

This is a correction to: Subhadra Evans, David Skvarc, Adrian Esterman, Matthew I Mackay, Melissa O’Shea, Leesa Van Niekerk, Marilla L Druitt, Jim Tsaltas, Simon R Knowles, Elesha Parigi, Katherine Stanley, Jill Harris, Meg Barber, Madeleine Dober, Charlotte Dowding, Antonina Mikocka-Walus, Telehealth cognitive behavioural therapy improves health-related quality of life and pain in endometriosis: the Healing Pelvic Pain Intervention (HaPPI)—a randomized controlled trial, *Human Reproduction Open*, Volume 2026, Issue 1, 2026, hoag006, https://doi.org/10.1093/hropen/hoag006.

In the originally published version of this manuscript, the duration of the yoga sessions was erroneously listed as “Weekly group yoga sessions of 120 minutes for 8 weeks, delivered via Zoom” within the Materials and methods section.

This has been corrected to read: “Weekly group yoga sessions of 60 minutes for 8 weeks, delivered via Zoom”.

The authors would like to assure readers that this typographical error does not affect any results, conclusions, or other content of the article.

